# Glomerular filtration rate estimated by differing measures and risk of all‐cause mortality among Chinese individuals without or with diabetes: A nationwide prospective study

**DOI:** 10.1111/1753-0407.13393

**Published:** 2023-05-01

**Authors:** Yu‐Jie Liu, Fu‐Rong Li, Cai‐Long Chen, Zhong‐Xiao Wan, Jin‐Si Chen, Jing Yang, Rong Liu, Jia‐Ying Xu, Yan Zheng, Li‐Qiang Qin, Guo‐Chong Chen

**Affiliations:** ^1^ Department of Nutrition and Food Hygiene, School of Public Health Suzhou Medical College of Soochow University Suzhou China; ^2^ School of Public Health and Emergency Management Southern University of Science and Technology Shenzhen China; ^3^ Children Health Management Center Children's Hospital of Soochow University Suzhou China; ^4^ Department of Clinical Nutrition The First Affiliated Hospital of Soochow University Suzhou China; ^5^ Department of Endocrine Changzhou Geriatric Hospital Affiliated to Soochow University Changzhou China; ^6^ State Key Laboratory of Radiation Medicine and Protection, School of Radiation Medicine and Protection Suzhou Medical College of Soochow University Suzhou China; ^7^ State Key Laboratory of Genetic Engineering, Human Phenome Institute, and School of Life Sciences Fudan University Shanghai China; ^8^ Ministry of Education Key Laboratory of Public Health Safety, School of Public Health Fudan University Shanghai China; ^9^ National Clinical Research Center for Aging and Medicine, Huashan Hospital Fudan University Shanghai China

**Keywords:** all‐cause mortality, chronic kidney disease, creatinine, cystatin C, diabetes, estimated glomerular filtration rate, 全因死亡率, 慢性肾病, 肌酐, 胱抑素C, 糖尿病, 估计肾小球滤过率

## Abstract

**Background:**

Whether estimated glomerular filtration rates (eGFRs) by differing biomarkers are differentially associated with mortality or whether the associations differ by diabetes status remains unclear, especially in Chinese population.

**Methods:**

We included 6995 participants without diabetes (mean age: 60.4 years) and 1543 with diabetes (mean age: 61.8 years). Each eGFR measure was divided into normal (≥90 mL/min/1.73 m^2^), modestly declined (60 to <90 mL/min/1.73 m^2^), and chronic kidney disease (CKD) (<60 mL/min/1.73 m^2^) groups. Cox proportional hazards models were used to estimate hazard ratio (HR) of all‐cause mortality associated with each eGFR.

**Results:**

Over a follow‐up of 7 years, 677 and 215 deaths occurred among individuals without or with diabetes, respectively. Among those without diabetes, all measures of modestly declined eGFR were not associated with mortality, whereas CKD defined by eGFR cystatin C (eGFRcys) and eGFR creatinine (eGFRcr)‐cys (HRs were 1.71 and 1.55, respectively) but not by eGFRcr were associated with higher risk of mortality. Among diabetes, all measures of modestly declined eGFR (HRs: 1.53, 1.56, and 2.09 for eGFRcr, eGFRcys, and eGFRcr‐cys, respectively) and CKD (HRs: 2.57, 2.99, and 3.92 for eGFRcr, eGFRcys, and eGFRcr‐cys, respectively) were associated with higher risk of mortality. Regardless of diabetes status, an addition of eGFRcys or eGFRcr‐cys to traditional risk factors lead to a larger improvement in the prediction of all‐cause mortality risk than adding eGFRcr.

**Conclusions:**

The association of eGFR with mortality risk appeared to be varied by its measures and by diabetes status among middle‐aged and older Chinese, which needs to be considered in clinical practice.

## Graphical abstract

**Highlights**

- All three estimated glomerular filtration rates (GFRs) (based on creatinine [eGFRcr], cystatin C [eGFRcys], or both [eGFRcr‐cys]) were more strongly associated with risk of all‐cause mortality among individuals with diabetes than among those without diabetes.
- Regardless of diabetes status, eGFRcys and eGFRcr‐cys were more strongly associated with risk of all‐cause mortality than eGFRcr.
- Regardless of diabetes status, an addition of eGFRcys or eGFRcr‐cys to traditional risk factors lead to a larger improvement in the prediction of all‐cause mortality risk than adding eGFRcr, which needs to be considered in clinical practices.

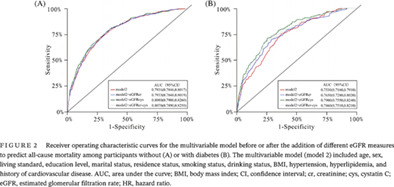

## 摘要

**【摘要】 背景** 通过不同生物标志物估计的肾小球滤过率(eGFRs)是否与死亡率不同相关，或者这种相关性是否因糖尿病状态而不同仍然不清楚，特别是在中国人群中。**方法** 本研究纳入了6 995名非糖尿病患者(平均年龄60.4岁)和1 543例糖尿病患者(平均年龄61.8岁)。每项eGFR测量分为正常[≥90 mL/min^‐1^·(1.73 m^2^)^‐1^]、中度下降[ 60~90 mL/min^‐1^·(1.73 m^2^)^‐1^]和慢性肾病(CKD) [ <60 mL/min^‐1^·(1.73 m^2^)^‐1^]组。*Cox*比例风险模型用于估计与每种eGFR相关的全因死亡率的风险比(*HR*)。**结果** 在7年的随访中，非糖尿病患者有677人死亡，糖尿病患者有215人死亡。在非糖尿病的患者中，eGFR轻度下降的所有指标都与死亡率无关，而由eGFRcys和eGFRcr‐cys定义的CKD(*HR*分别为1.71和1.55)，而不是由eGFRcr定义的CKD与较高的死亡风险相关。在糖尿病患者中，所有适度下降的eGFR(分别为eGFRcr、eGFRcys和eGFRcr‐cys的*HR* 1.53、1.56和2.09)和CKD(分别为eGFRcr、eGFRcys和eGFRcr‐cys的*HR* 2.57、2.99和3.92)与较高的死亡风险相关。无论糖尿病状态如何，在传统的危险因素中加入eGFRcys或eGFRcr‐cys，比加入eGFRcr，在预测全因死亡风险方面有更大的改善。**结论** 在中国老年人群中，eGFR与死亡风险的关系似乎因其测量方法和糖尿病状况而异，这需要在临床实践中加以考虑。

## 1 INTRODUCTION

Chronic kidney disease (CKD), a condition that causes a gradual decline in kidney function over time, is an important and increasing global public health problem affecting 9.1% of adults worldwide.^1^, ^2^ In China, the national prevalence of CKD was estimated to be 10.8%, imposing health and financial hardships to the society.^3^

Estimated glomerular filtration rate (eGFR) is regularly used in clinical practices to characterize kidney function and define CKD, with the most popular one being creatinine‐based eGFR (eGFRcr). Notably, serum creatinine is not only affected by health status but also by other host factors such as age, muscle mass, and diet.^4^ Serum cystatin C‐based eGFR (eGFRcys) has been recommended as an alternate filtration marker to estimate renal function.^5^ A recent meta‐analysis of 16 studies (including 11 general‐population studies and 5 studies of individuals with CKD) found that eGFRcys had a stronger association with all‐cause mortality than eGFRcr.^6^ However, non‐GFR determinants, such as body mass index (BMI), diabetes, and inflammation, also affect the accuracy of eGFRcys in assessing kidney function,^7^, ^8^ such that this measure may perform differentially under different demographic and clinical conditions. Given these potential limitations of both eGFRcys and eGFRcr, recent studies have used a combination of creatinine and cystatin C (eGFRcr‐cys) and suggested that this measure could more accurately reflect kidney function than the GFR estimated by either marker alone.^9^, ^10^

Among individuals with diabetes, CKD is one of the most common complications and has been widely associated with increased risk of premature death.^11^, ^12^, ^13^ The relationship between declined kidney function or CKD and mortality among the general population appeared to be weaker and less consistent.^6^ Previous studies on multiple eGFR measures and mortality did not clearly distinguish between individuals with diabetes and those without, and so it remains unclear whether eGFR equations based on creatinine, cystatin C, or both biomarkers are similarly predictive of mortality risk regardless of diabetes status.^14^, ^15^, ^16^

To fill the knowledge gaps, we used data from the China Health and Retirement Longitudinal Study (CHARLS), a nationally representative cohort study of middle‐aged and older Chinese adults, to investigate the relationship between eGFR estimated by creatinine, cystatin C, or a combination of both and all‐cause mortality according to diabetes status.

## 2 METHODS

### 2.1 Study design and population

The CHARLS is a population‐based prospective cohort study of individuals aged 45 years and upwards and is currently ongoing in China. Participants were recruited using a multistage probability sampling procedure from 150 counties across 30 provincial administrative units.^17^ The first wave (W1) examination of participants was performed in 2011–2012, and data on demographic, socioeconomic, lifestyle, physical measures, and health‐related information were obtained. Three additional follow‐up visits were conducted in 2013 (W2), 2015 (W3), and 2018 (W4), respectively. A further detailed description of the study rationale, design, and participant characteristics for the CHARLS has been previously published.^17^ The project was approved by the Ethical Committee of Peking University and signed informed consents were obtained from all participants.

This study used data from the four waves of the CHARLS. To be included in the current analysis, the study participants had to meet the following criteria: (a) had measurements of serum creatinine and cystatin C levels at W1; (b) had information to define diabetes status at W1; (c) were aged 45 years or older at W1; and (d) were successfully followed up in at least one of the next three waves. Finally, 8538 participants were included for further investigation (Figure S1 in Data S1).

### 2.2 Laboratory assays

For each participant, venous blood (over 92% were fasting) was taken by trained personnel and delivered to a nearby laboratory at 4°C. Whole blood and centrifuged serum samples were frozen at −20°C, delivered to the Chinese Center for Disease Control and Prevention within 2 weeks in Beijing, and stored at −80°C until evaluated in the laboratory of Capital Medical University. The boronated affinity HPLC was used to test glycated hemoglobin (HbA1c). Serum cystatin C and creatinine were measured at baseline (W1) using a particle‐enhanced turbimetric assay with a coefficient of variation (CV) of 5.0% between assays and a rate‐blanked and compensated Jaffe creatinine technique with a CV of 2.1% between assays, respectively. Serum major lipids including high‐density lipoprotein cholesterol, low‐density lipoprotein cholesterol, triglycerides, and plasma glucose were all determined using an enzymatic colorimetric assay. The CHARLS research samples underwent quality control (QC). All QC sample test findings were within 2 SDs of the mean QC control concentrations. Details of the laboratory analyses have been reported elsewhere.^18^, ^19^

### 2.3 Assessment kidney function

We used three eGFRs to assess kidney function. The Chronic Kidney Disease Epidemiology Collaboration (CKD–EPI) formulas were used to measure eGFRs considering serum creatinine, cystatin C, or a combination of both, in addition to age and sex (Table S1 in Data S1).^4^, ^20^ Each eGFR measure was then divided into normal (≥90 mL/min/1.73 m^2^), modestly declined (60 to <90 mL/min/1.73 m^2^), and CKD (<60 mL/min/1.73 m^2^) groups.

### 2.4 Assessment of diabetes and other covariates

Information on various sociodemographic and lifestyle factors, such as age, sex, living standard, education level, marital status, residence status (urban/rural), current smoking status, alcohol drinking (drink more than once a month, drink but less than once a month, none of these), was obtained by interviewer‐administered questionnaires. Respondents were asked: ‘How is your standard of living?’ Five responses were provided: very high, relatively high, average, relatively poor, and poor. Living standard was further divided into high (very high or relatively high), average, and poor (relatively poor or poor) groups. BMI was calculated as measured weight (kg) divided by measured height (m^2^). Diabetes was considered present if participants were diagnosed with diabetes by a physician or had fasting plasma glucose ≥126 mg/dL or HbA1c ≥6.5% at the W1 survey. Hypertension was defined as systolic blood pressure ≥140 mm Hg, diastolic blood pressure ≥90 mm Hg, or self‐report of physician‐diagnosed hypertension or use of antihypertensive drugs. In formation on diagnoses of other medical conditions such as hyperlipidemia, heart problems (including heart attack, coronary heart disease, angina, congestive heart failure, or other heart problems) and stroke was also collected.

### 2.5 All‐cause mortality follow‐up

Participants enrolled in W1 were followed up in the subsequent three waves. Detailed vital status and date of death (for those who were deceased) were available in W2, whereas only vital status was recorded in W3 and W4. For participants who were recorded as being decreased at W2 survey, follow‐up time was the interval between W1 interview and the date of death. For participants who were recorded as being deceased at W3, follow‐up time was the interval time between W1 and W2 in addition to median of the period between W2 and W3. A similar approach was used to derive follow‐up time for participants who were recorded as being deceased at W4 (W1–W3 period plus median of W3–W4). For the remaining individuals, follow‐up time was defined as the interval between W1 and the last interview wave with follow‐up information.

### 2.6 Statistical analysis

Baseline participant characteristics were reported by eGFRcr‐cys classifications (normal, modestly declined, and CKD), separately for participants without or with diabetes. Continuous variables were represented as mean ± SDs and categorical variables as numbers (percentages). Where appropriate, the analysis of variance or Kruskal–Wallis tests were used to assess continuous variables, and the chi‐square or Fisher's exact tests were used for categorical variables.

Multivariate Cox proportional hazard models were used to examine the relationship between eGFRs and all‐cause mortality among individuals without or with diabetes, with the normal group as the reference. Two models with different degrees of adjustment for potential confounders were created. Model 1 was adjusted for age and sex. Model 2 was adjusted for age and sex and additionally adjusted for living standard, education level, marital status, residence status, smoking status, drinking status, BMI, hypertension, hyperlipidemia, and history of cardiovascular disease (ie, heart problems or stroke). To assess the potential nonlinearity for the examined relationships, each eGFR measure as a continuous variable was further modeled using restricted cubic splines with five knots at 5th, 27.5th, 50th, 72.5th, and 95th percentiles of the distribution of each eGFR.

We performed a sensitivity analysis to examine different eGFRs in relation to risk of all‐cause mortality after excluding participants with eGFR of <15 or ≥120 mL/min/1.73m^2^. In addition, among participants without diabetes, we performed a further analysis to examine different eGFR measures in relation to all‐cause mortality by the status of prediabetes at baseline. Moreover, among participants who had normal kidney function defined by eGFRcr (≥90 mL/min/1.73m^2^), we examined the relationship between the other two eGFR measures (eGFRcys and eGFRcr‐cys) and risk of all‐cause mortality using the aforementioned full model.

Finally, we evaluated improvements in the prediction of all‐cause mortality risk after adding each of the eGFRs to the multivariable model including traditional risk factors. Model discrimination and reclassification were evaluated using areas under receiver operating characteristic curves (AUCs), continuous net reclassification improvement indices, integrated discrimination improvement index, and change in C index.^21^ Statistical analyses were performed using R software and SAS version 9.4. A two‐sided *p* value of <.05 was considered statistically significant.

## 3 RESULTS

### 3.1 Baseline participant characteristics

There were 6995 participants without diabetes and 1543 with diabetes in the final analysis. Mean age was 60.4 years among those without diabetes (46.5% were aged 60 years or older), and 61.8 years among those with diabetes (53.1% were aged 60 years or older). Regardless of diabetes status, participants with lower eGFRcr‐cys were older; were more likely to be male, smokers, and regular drinkers; had higher systolic blood pressure; and had lower triglycerides, BMI, and educational levels. Lower eGFRcr‐cys was also associated with higher likelihood of cardiovascular disease and hypertension (Table 1).

**TABLE 1:** Baseline participant characteristics according to eGFRcr‐cys groups among individuals without or with diabetes (*n* = 8538).

Variables	Without diabetes (*n* = 6995)				With diabetes (*n* = 1543)			
eGFRcr‐cys, mL/min/1.73m^2^	<60	60 to <90	≥90	*p* value	<60	60 to <90	≥90	*p* value
*N*	634	3732	2629		189	768	586	
Age (years)	72.4 ± 9.1	62.6 ± 9.1	54.4 ± 7.2	<.001	71.6 ± 8.1	63.7 ± 9.1	56.3 ± 7.7	<.001
Male sex, %	51.3	49.9	44.3	<.001	50.3	47.9	43.7	.17
Education, %				<.001				<.001
No formal education	64.8	51.9	41.3		59	52	44.9	
Primary school or middle school	29.5	38.8	45.8		34	38.1	41	
High school or above	5.7	9.3	12.9		6.9	9.9	14.2	
Current smoker, %	45.4	42.7	35.4	<.001	36.6	41.4	34.5	.034
Drinking, %				<.001				.020
Drink more than once a month	76.8	66.6	64.9		74.7	73.5	66.7	
Drink but less than once a month	4.6	8	7.3		4.3	5.9	8.6	
None of these	18.6	25.3	27.7		21	20.6	24.8	
Glycated hemoglobin (%)	5.1 ± 0.4	5.1 ± 0.4	5.1 ± 0.4	.022	5.9 ± 1.4	6.0 ± 1.4	6.2 ± 1.7	.087
SBP (mm Hg)	141.8 ± 33.1	132.1 ± 23.9	126.9 ± 23.8	<.001	143.6 ± 21.3	138.5 ± 28.4	132.9 ± 29.3	<.001
DBP (mm Hg)	75.1 ± 12.5	75.4 ± 12.3	75.3 ± 12.0	.86	77.4 ± 12.9	77.2 ± 11.7	77.4 ± 11.7	.91
LDL cholesterol (mmol/L)	117.1 ± 34.9	118.1 ± 34.2	115.6 ± 33.6	.016	116.6 ± 42.1	114.0 ± 38.1	114.9 ± 40.1	.87
HDL cholesterol (mmol/L)	52.2 ± 14.7	52.7 ± 15.4	51.4 ± 14.7	<.001	46.8 ± 14.9	46.9 ± 16.1	44.9 ± 15.4	.028
Creatinine (mg/dL)	1.1 ± 0.6	0.8 ± 0.2	0.7 ± 0.1	<.001	1.1 ± 0.3	0.8 ± 0.2	0.7 ± 0.1	<.001
Cystatin C (mg/L)	1.6 ± 0.5	1.1 ± 0.1	0.8 ± 0.1	<.001	1.6 ± 0.4	1.1 ± 0.1	0.8 ± 0.1	<.001
BMI (kg/m^2^), %				<.001				<.001
<18.5	28.1	24.1	21.3		27.8	23	22.1	
18.5 ≤ 22.5	35.4	35.1	31.3		31.8	25	20.3	
22.5 ≤ 25	20.2	21.4	23.5		19.7	20.6	25.7	
25 ≤ 27.5	11.4	12.8	15.8		13.9	17.7	19.2	
>27.5	4.9	6.7	8.2		6.9	13.7	12.8	
Hypertension, %	59.6	40.6	29.9	<.001	67.7	55.1	45.6	<.001
Hyperlipidemia, %	7.4	7.5	7.5	.90	21.7	17.0	16.2	.060
Cardiovascular disease, %	19.4	15.8	9.4	<.001	31	21	18.1	<.001

*Note*: Data are expressed as the mean ± SD or *n* (%).

Abbreviations: BMI, body mass index; DBP, diastolic blood pressure; eGFRcr‐cys, estimated glomerular filtration rate based on a combination of creatinine and cystatin C; eGFRcr, estimated glomerular filtration rate based on serum creatinine; eGFRcys, estimated glomerular filtration rate based on serum cystatin C; HDL cholesterol, high‐density lipoprotein cholesterol; LDL cholesterol, low‐density lipoprotein cholesterol; SBP, systolic blood pressure.

### 3.2 Associations between different eGFRs and risk of all‐cause mortality by diabetes status

Over a follow‐up of 7 years, 677 deaths occurred among the 6995 participants without diabetes at baseline, and 215 deaths occurred among the 1543 participants with diabetes. There were significant interactions between each of the eGFR measures and diabetes status on risk of all‐cause mortality (*p*‐for‐interaction values: .04, .02, and .03 for eGFRcr, eGFRcys, and eGFRcr‐cys, respectively). Thus, the associations between different eGFR measures and risk of all‐cause mortality were reported according to diabetes status.

Among participants without diabetes, there were significant associations between CKD (eGFR<60 mL/min/1.73m^2^) defined by eGFRcys or eGFRcr‐cys and higher risk of mortality, with fully adjusted HRs of 1.71 (95% CI: 1.28–2.28) and 1.55 (95% CI: 1.17–2.07), respectively. CKD defined by eGFRcr was not significantly associated with risk of all‐cause mortality (HR = 1.16; 95% CI: 0.86–1.56). Regarding eGFR measures, modestly declined eGFR (60 to <90 mL/min/1.73m^2^) was not associated with risk of all‐cause mortality among participants without diabetes (Table 2).

**TABLE 2:** The association between different eGFR measures and risk of all‐cause mortality among participants without diabetes.

eGFR, mL/min/1.73m^2^	*N*	Events	Unadjusted	Model 1	Model 2
			HR (95% CI)	HR (95% CI)	HR (95% CI)
eGFRcr categories					
≥90	4204	262	Ref	Ref	Ref
60 to <90	2523	342	2.32 (1.97–2.72)	0.87 (0.72, 1.04)	0.87 (0.72–1.06)
<60	268	73	5.12 (3.95–6.65)	1.06 (0.79, 1.43)	1.16 (0.86–1.56)
*p* for trend			<.0001	.75	.89
Per 10‐unit decrease			1.41 (1.35–1.47)	1.03 (0.97, 1.09)	1.04 (0.98–1.10)
eGFRcys categories					
≥90	1933	76	Ref	Ref	Ref
60 to <90	3587	263	1.91 (1.48–2.48)	1.15 (0.88, 1.49)	1.12 (0.60–1.47)
<60	1475	338	6.52 (5.01–8.38)	1.71 (1.29, 2.26)	1.71 (1.28–2.28)
*p* for trend			<.0001	<.0001	<.0001
Per 10‐unit decrease			1.48 (1.43–1.54)	1.14 (1.09, 1.19)	1.14 (1.09–1.19)
eGFRcr‐cys categories					
≥90	2629	110	Ref	Ref	Ref
60 to <90	3732	389	2.61 (2.11–3.23)	1.16 (0.93, 1.46)	1.14 (0.90–1.44)
<60	634	178	8.12 (6.40–10.32)	1.55 (1.17, 2.06)	1.55 (1.17–2.07)
*p* for trend			<.0001	.0015	.0015
Per 10‐unit decrease			1.51 (1.45–1.57)	1.13 (1.07, 1.19)	1.13 (1.07–1.20)

*Note*: Model 1: adjusted for age and sex. Model 2: adjusted for age, sex, living standard, education level, marital status, residence status, smoking status, drinking status, BMI, hypertension, hyperlipidemia, and history of cardiovascular disease.

Abbreviations: BMI, body mass index; CI, confidence interval; eGFRcr‐cys, estimated glomerular filtration rate based on a combination of creatinine and cystatin C; eGFRcr, estimated glomerular filtration rate based on serum creatinine; eGFRcys, estimated glomerular filtration rate based on serum cystatin C.

Among participants with diabetes, CKD was substantially associated with higher risk of all‐cause mortality regardless of eGFR measures, with fully adjusted HRs of 2.57 (95% CI: 1.62–4.07) for eGFRcr, 2.99 (95% CI: 1.83–4.88) for eGFRcys, and 3.92 (95% CI: 2.35–6.53) for eGFRcr‐cys defined CKD. Modestly declined eGFRcr (HR = 1.53; 95% CI: 1.08–2.16), eGFRcys (HR = 1.56, 95% CI: 0.97–2.49), and eGFRcr‐cys (HR = 2.09; 95% CI: 1.34–3.28) were also associated with higher risk all‐cause mortality, though the risk estimate for eGFRcys was not statistically significant (Table 3).

**TABLE 3:** The association between different eGFR measures and risk of all‐cause mortality among participants with diabetes.

eGFR, mL/min/1.73m^2^	*N*	Events	Unadjusted	Model 1	Model 2
			HR (95% CI)	HR (95% CI)	HR (95% CI)
eGFRcr categories					
≥90	841	63	Ref	Ref	Ref
60 to <90	594	114	2.77 (2.04–3.77)	1.47 (1.05, 2.07)	1.53 (1.08–2.16)
<60	108	38	5.68 (3.80–8.50)	2.14 (1.36, 3.37)	2.57 (1.62–4.07)
*p* for trend			<.0001	.0009	<.0001
Per 10‐unit decrease			1.37 (1.28–1.46)	1.12 (1.03, 1.22)	1.16 (1.07–1.27)
eGFRcys Categories					
≥90	478	25	Ref	Ref	Ref
60 to <90	709	79	2.17 (1.39–3.41)	1.60 (1.01, 2.52)	1.56 (0.97–2.49)
<60	356	111	6.90 (4.47–10.65)	2.89 (1.79, 4.67)	2.99 (1.83–4.88)
*p* for trend			<.0001	<.0001	<.0001
Per 10‐unit decrease			1.46 (1.37–1.55)	1.26 (1.17, 1.36)	1.30 (1.20–1.41)
eGFRcr‐cys categories					
≥90	586	27	Ref	Ref	Ref
60 to <90	768	119	3.54 (2.33–5.38)	2.19 (1.41, 3.38)	2.09 (1.34–3.28)
<60	189	69	9.43 (6.04–14.71)	3.67 (2.22, 6.04)	3.92 (2.35–6.53)
*p* for trend			<.0001	<.0001	<.0001
Per 10‐unit decrease			1.48 (1.38–1.58)	1.25 (1.16, 1.36)	1.31 (1.20–1.42)

*Note*: Model 1: adjusted for age and sex. Model 2: adjusted for age, sex, living standard, education level, marital status, residence status, smoking status, drinking status, BMI, hypertension, hyperlipidemia, and history of cardiovascular disease.

Abbreviations: BMI, body mass index; CI, confidence interval; eGFRcr‐cys, estimated glomerular filtration rate based on a combination of creatinine and cystatin C; eGFRcr, estimated glomerular filtration rate based on serum creatinine; eGFRcys, estimated glomerular filtration rate based on serum cystatin C.

**FIGURE 1:**
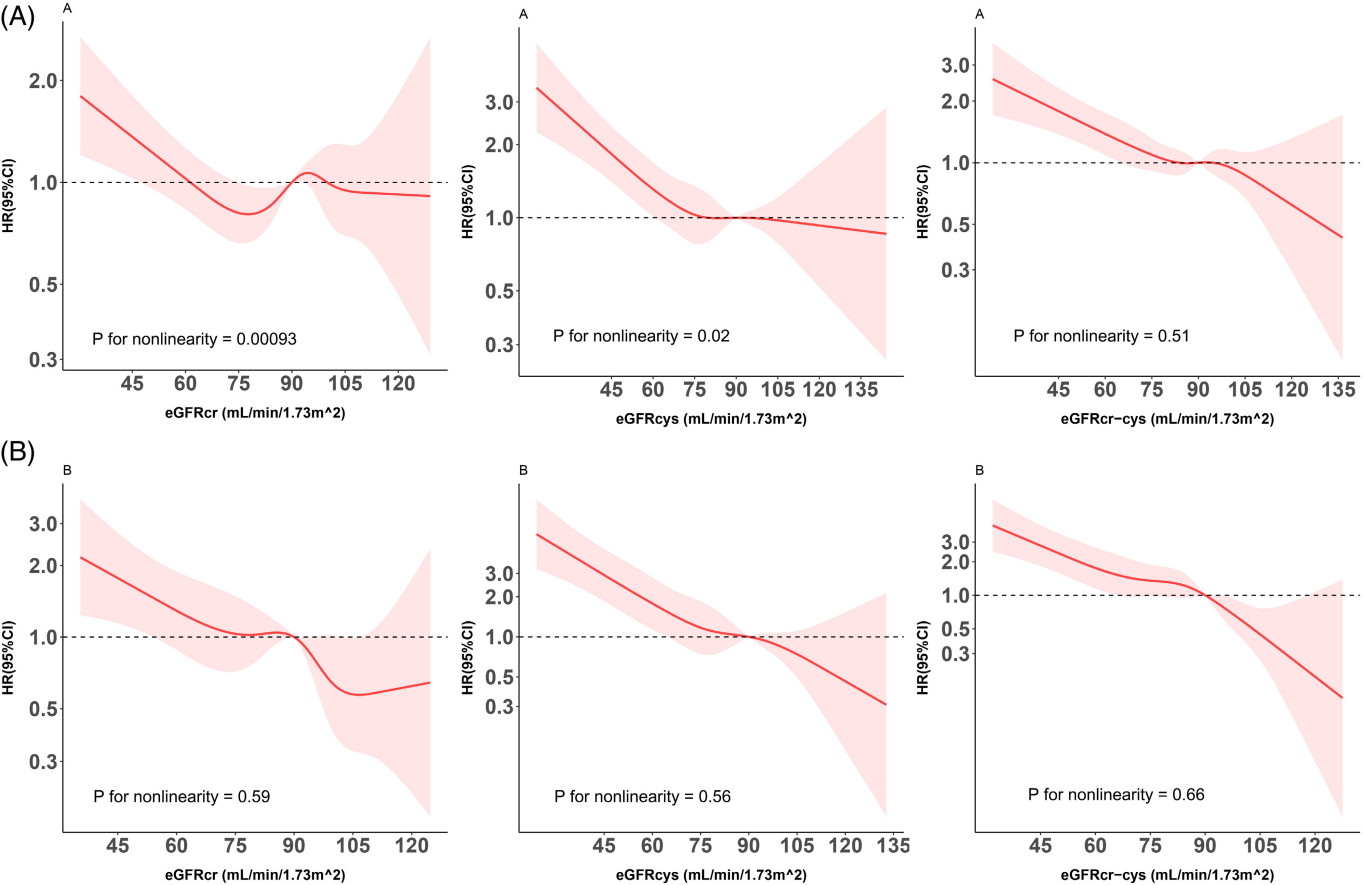
Restricted cubic splines for the association of different eGFR measures among individuals without (panel A) or with diabetes (panel B). The splines were modeled with five knots (5th, 27.5th, 50th, 72.5th, and 95th percentiles), and the level of 90 mL/min/1.73 m^2^ was used as the reference. Results were adjusted for age, sex, living standard, education level, marital status, residence status, smoking status, drinking status, BMI, hypertension, hyperlipidemia, and history of cardiovascular disease. BMI, body mass index; CI, confidence interval; cr, creatinine; cys, cystatin C; eGFR, estimated glomerular filtration rate; HR, hazard ratio.

Among those without diabetes, evidence for a nonlinear (L‐shaped) relationship was observed between eGFRcr (*p* for nonlinearity <.001) or eGFRcys (*p* for nonlinearity = .023) and risk of all‐cause mortality, while the association for eGFRcr‐cys was more linear (*p* for nonlinearity = .51) (Figure 1). For all three eGFR measures, their relationships with all‐cause mortality were monotonously linear among participants with diabetes (*p* values for nonlinearity >.50).

### 3.3 Sensitivity analyses

For each of the eGFRs, the association with risk of all‐cause mortality was similar after excluding a small number of participants with the corresponding eGFR of <15 or ≥120 mL/min/1.73m^2^ (Tables S2 and S3 in Data S1).

There were 4204 participants without diabetes and 841 participants with diabetes who did not have CKD according to eGFRcr, among whom we further evaluated eGFRcys and eGFRcr‐cys in relation to mortality risk. There were significant associations between modestly declined eGFR defined by eGFRcys or eGFRcr‐cys and increased risk of all‐cause mortality among participants with diabetes but not among those without diabetes (Tables S4 and S5 in Data S1). CKD defined by eGFRcys was associated with higher mortality risk regardless of diabetes status, and there were too few deaths for the analysis of CKD defined by eGFRcr‐cys in this subgroup of population.

Among the 6995 participants without diabetes, there were 3664 participants with prediabetes. In respective of prediabetes status, modestly declined eGFR defined by any of the three measures were not associated with risk of mortality, whereas CKD defined by eGFRcys or eGFRcr‐cys but not by eGFRcr was associated with higher risk of all‐cause mortality (Tables S6 and S7 in Data S1).

### 3.4 Predictive value of each eGFR measure for all‐cause mortality

**FIGURE 2:**
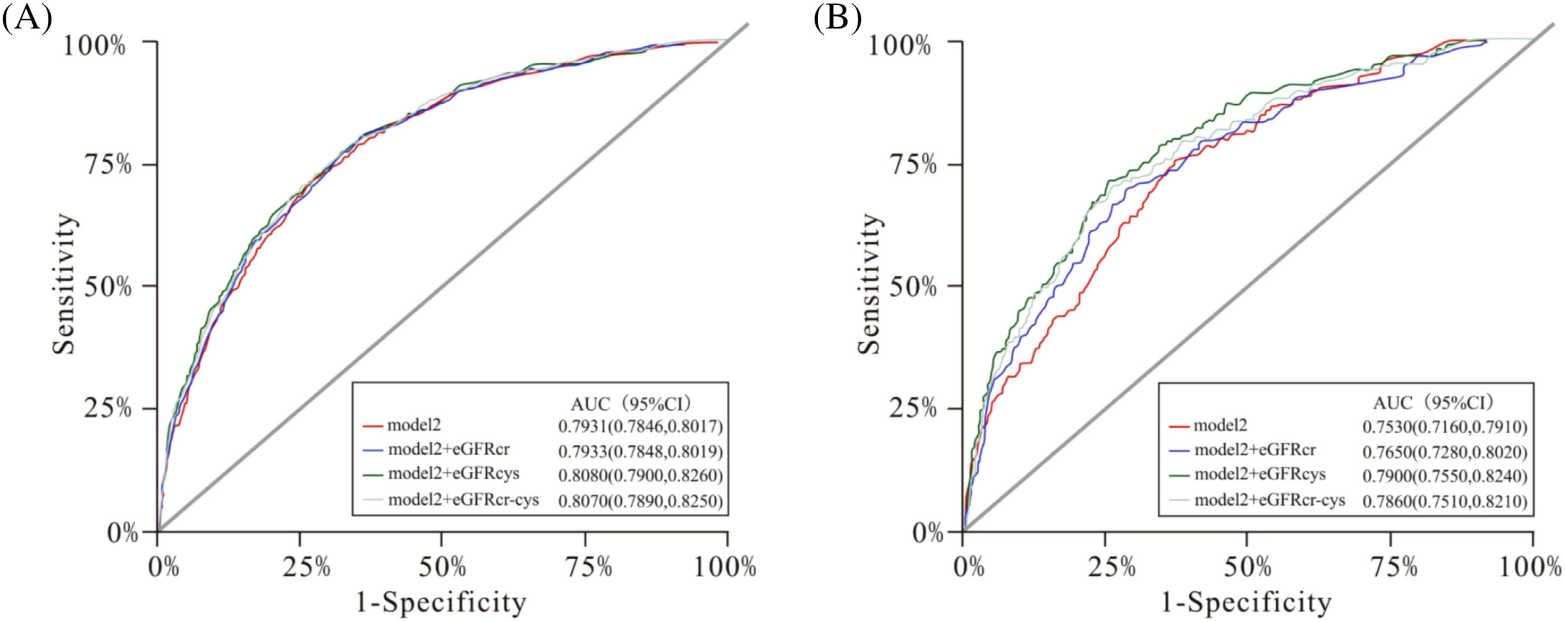
Receiver operating characteristic curves for the multivariable model before or after the addition of different eGFR measures to predict all‐cause mortality among participants without (A) or with diabetes (B). The multivariable model (model 2) included age, sex, living standard, education level, marital status, residence status, smoking status, drinking status, BMI, hypertension, hyperlipidemia, and history of cardiovascular disease. AUC, area under the curve; BMI, body mass index; CI, confidence interval; cr, creatinine; cys, cystatin C; eGFR, estimated glomerular filtration rate; HR, hazard ratio.

Among individuals with diabetes, an addition of eGFRcr to the multivariable model (model 2) including traditional factors increased the AUC from 0.7530 (95% CI: 0.7160–0.7910) to 0.7650 (95% CI: 0.7280–0.8020), and the AUC was slightly higher when eGFRcys (AUC: 0.7900; 95% CI: 0.7550–0.8240) or eGFRcr‐cys (AUC: 0.7860; 95% CI: 0.7510–0.8210) was added to the multivariable model (Figure 2). Differences in AUC were smaller among participants without diabetes. Further analyses of changes in C index and continuous net reclassification improvement indices and integrated discrimination improvement index supported these results (Table S8 in Data S1).

## 4 DISCUSSION

In a nationally representative sample of middle‐aged and older Chinese, we used validated eGFR prediction equations to examine the associations of three eGFR measures based on creatinine, cystatin C, or both biomarkers with all‐cause mortality according to diabetes status. Our study has several major findings. First, our findings showed that the relationship between eGFR and mortality risk appeared to be dependent on its measures. There was a weaker association between eGFRcr and mortality than the associations for other two eGFR measures (eGFRcys or eGFRcr‐cys) regardless of diabetes status. Second, for each of the three eGFR measures, the relationship with mortality risk appeared to be modified by diabetes status, with substantially stronger associations among participants with diabetes than among those without. Third, an addition of eGFRcys or eGFRcr‐cys into the multivariable model that included traditional risk factors improved predictive value for risk of mortality, especially so among participants with diabetes.

Although the CKD‐EPI formula^4^ has been commonly used to investigate the association of eGFR with mortality, the majority of previous studies have been focused on the general population.^22^, ^23^, ^24^, ^25^ A few studies have compared eGFR based on creatinine or cystatin C for the association with all‐cause mortality among individuals with diabetes. For example, in the Fukuoka Diabetes Registry including 4869 participants aged 20 years or older who were followed up for a mean of 3.3 years, there was a significant association between eGFRcr or eGFRcys and all‐cause mortality at the eGFR range of ≤29 mL/min/1.73m^2^, with fully adjusted HRs of 2.43 (95% CI: 1.16–5.08) and 5.77 (95% CI: 2.77–12.03), respectively.^26^ Similarly, findings from the 1999–2002 National Health and Nutrition Examination Survey (NHANES) showed that eGFRcys was more strongly associated with all‐cause mortality risk than eGFRcr among individuals with diabetes.^27^ Despite using different eGFR cutoffs in the analyses, these findings are consistent with our results on the association between eGFR based on creatinine or cystatin C and all‐cause mortality among participants with diabetes. Notably, in our study population, even modestly declined kidney function (eGFR: 60 to <90 mL/min/1.73m^2^) was associated with increased risk of all‐cause mortality among individuals with diabetes.

Our study extended the previously reported associations in diabetes to a Chinese population by diabetes status, showing that each of the eGFR measures was more strongly associated with mortality risk among individuals with diabetes than among those without diabetes. Nevertheless, our findings further showed that eGFR based on cystatin C or a combination of creatinine and cystatin C was more predictive of mortality risk than eGFRcr regardless of diabetes status. In addition, even among individuals without CKD according to eGFRcr, both eGFRcys and eGFRcr‐cys remained significantly and positively associated with risk of all‐cause mortality (Tables S4 and S5 in Data S1). Furthermore, among individuals without diabetes, CKD defined by eGFRcr was not associated with mortality risk. These findings highlight the importance of considering both eGFR measures and health conditions (eg, diabetes status) in clinical practices.

Given that the estimating equation incorporating both creatinine and cystatin C is more positively related with directly measured GFR than each of other two eGFR measures,^28^ it would be expected that the combined equation could be most strongly associated with risk of mortality. We observed, however, similar strength of associations between eGFR estimated with cystatin C alone or in combination with creatinine and risk of mortality. In another analysis of US participants aged 18 years or older in the NHANES III, Astor et al^29^ found that the association between eGFR based on both creatinine and cystatin C was a weak predictive marker when compared to cystatin C. The reasons for the differences in previous findings and ours might be differences in age, racial/ethnic groups, or other participant characteristics, and future studies, especially those that distinguish between individuals with and without diabetes, are still required to confirm our results.

Potential explanations for the differences in the relationship between eGFR and mortality risk by different eGFR measures remain unclear. Previous studies have shown that serum creatinine is widely affected by age, muscle mass, diet, and physical activity, particularly in the high‐to‐normal range, and thus may be of limited abilities to identify early renal impairment.^6^, ^27^, ^30^, ^31^, ^32^ On the other hand, cystatin C is less affected by age and muscle mass and appears to be more sensitive than creatinine in detecting mild‐to‐moderate renal impairment.^33^, ^34^, ^35^, ^36^ In addition, cystatin C has been shown to be more accurate at detecting a rapid decline in GFR than creatinine measurements among participants with type 1 diabetes who have normal GFR levels.^37^ Participants in the CHARLS are relatively older (mean age: 60.7 years) and may have reduced muscle mass, which may have affected the ability of the eGFR based on creatinine in characterizing kidney function.^38^ Our findings, together with findings from other previous epidemiologic studies,^39^, ^40^, ^41^ support the regular use of cystatin C in the assessment of kidney function in clinical practices. With the increasing availability of medical technology and diagnostic testing, the cost of cystatin C measurements has been reduced.^42^ This recognition of the value of cystatin C may lead to more adoption in clinical contexts in the future.

The present study is the first to evaluate the associations of eGFRs by different biomarkers and risk of all‐cause mortality according to diabetes status among Chinese. The current study has several strengths, which include a prospective design, a nationally representative sample, centralized measurements of blood samples in one single accredited laboratory, and comprehensive adjustment for the related covariates. We also included other comprehensive analyses in this study such as the metrics for multiple predictive models and the assessment of the potential nonlinearity for the examined associations. Notwithstanding, several potential limitations to this study should be considered. As an observational study, the potential influence of residual confounding on our results cannot be fully excluded. Due to the difficult procedures, adverse reactions, high cost, and the lack of urinary albumin, the GFR was estimated but not directly quantified.^43^ Additionally, because this study included only Chinese participants who were 45 years and older (47.7% were older than 60 years), generalization of the findings to younger or other racial/ethnic groups remains unclear.

## 5 CONCLUSIONS

In conclusion, our findings suggest that the relationship between eGFR and risk of all‐cause mortality appears to be different according to its measures and is modified by diabetes status. The eGFR measure based on cystatin C or both biomarkers (eGFRcys or eGFRcr‐cys) is more strongly associated with and more predictive of all‐cause mortality than the eGFR measure based on creatinine. Moreover, irrespective of eGFR measures, the relationship between eGFR and mortality risk is substantially stronger among participants with diabetes than among those without, which needs to be considered in clinical practices.

## FUNDING INFORMATION

This study was partially supported by Suzhou science and technology development plan (Livelihood Technology) project (No SS202009). Dr. Fu‐Rong Li is supported by Chinese Postdoctoral Science Foundation (2022M721463).

## CONFLICT OF INTEREST STATEMENT

The authors declare that they have no competing interests.

## Supporting information


**Data S1.** Supporting information.

## Data Availability

The CHARLS data sets, which were analyzed during the current study, are publicly available at the National School of Development, Peking University (http://charls.pku.edu.cn/en) and can be obtained after submitting a data use agreement to the CHARLS team.
