# Supplementary material for: Glomerular filtration rate estimated by differing measures and risk of all‐cause mortality among Chinese individuals without or with diabetes: A nationwide prospective study
Source: J Diabetes. 2023 May 1;15(6):508–18. doi: 10.1111/1753-0407.13393 (PMC10270745; doi:10.1111/1753-0407.13393)
Supplement: Supplementary file 1 — Data S1. Supporting information. [file JDB-15-508-s001.docx]

| **Supplementary Table 1. Creatinine equation (CKD-EPI 2009), cystatin C equation (CKD-EPI 2012), and creatinine–cystatin C**  **equation (CKD-EPI 2012) for estimating GFR, expressed for specified sex, serum creatinine level, and serum cystatin C level*** | | | |
| --- | --- | --- | --- |
| Basis of equation  and sex | Serum  creatinine | Serum  cystatin C | Equations for estimating GFR |
| CKD-EPI creatinine equation |  | | |
| Female | ≤0.7 |  | 144×(Scr/0.7) ^−0.329^×0.993 ^Age^ [× 1.159 if black] |
| Female | >0.7 |  | 144×(Scr/0.7) ^−1.209^×0.993 ^Age^ [× 1.159 if black] |
| Male | ≤0.9 |  | 141×(Scr/0.9) ^−0.411^×0.993 ^Age^ [× 1.159 if black] |
| Male | >0.9 |  | 141×(Scr/0.9) ^−1.209^×0.993 ^Age^ [× 1.159 if black] |
| CKD-EPI cystatin C equation |  | | |
| Female or male |  | ≤0.8 | 133×(Scys/0.8) ^−0.499^ × 0.996 ^Age^ [× 0.932 if female] |
| Female or male |  | >0.8 | 133×(Scys/0.8) ^−1.328^ × 0.996 ^Age^ [× 0.932 if female] |
| CKD-EPI creatinine-cystatin C equation |  | | |
| Female | ≤0.7 | ≤0.8 | 130×(Scr/0.7) ^−0.248^ ×(Scys/0.8) ^−0.375^ ×0.995 ^Age^ [× 1.08 if black] |
|  |  | >0.8 | 130×(Scr/0.7) ^−0.248^ ×(Scys/0.8) ^−0.711^ ×0.995 ^Age^ [× 1.08 if black] |
| Female | >0.7 | ≤0.8 | 130×(Scr/0.7) ^−0.601^ ×(Scys/0.8) ^−0.375^ ×0.995 ^Age^ [× 1.08 if black] |
|  |  | >0.8 | 130×(Scr/0.7) ^−0.601^ ×(Scys/0.8) ^−0.711^ ×0.995 ^Age^ [× 1.08 if black] |
| Male | ≤0.9 | ≤0.8 | 135×(Scr/0.9) ^−0.207^ ×(Scys/0.8) ^−0.375^ ×0.995 ^Age^ [× 1.08 if black] |
|  |  | >0.8 | 135×(Scr/0.9) ^−0.207^ ×(Scys/0.8) ^−0.711^ ×0.995 ^Age^ [× 1.08 if black] |
| Male | >0.9 | ≤0.8 | 135×(Scr/0.9) ^−0.601^ ×(Scys/0.8) ^−0.375^ ×0.995 ^Age^ [× 1.08 if black] |
|  |  | >0.8 | 135×(Scr/0.9) ^−0.601^ ×(Scys/0.8) ^−0.711^ ×0.995 ^Age^ [× 1.08 if black] |
| *****The information on race/ethnicity was not used in the current analysis of Chinese participants. | | | |

| **Supplementary Table 2. The association between different eGFR measures and risk of all-cause mortality among participants without diabetes who excluded those participants with each eGFR<15 and eGFR>120 mL/min/1.73m^2^** | | | | |
| --- | --- | --- | --- | --- |
| eGFR, mL/min/1.73m^2^ | N | Events | Model 1 | Model 2 |
|  |  |  | HR (95% CI) | HR (95% CI) |
| eGFRcr categories | 6255 | 667 |  |  |
| ≥90 | 3776 | 260 | Ref | Ref |
| 60 to <90 | 2252 | 342 | 0.89 (0.74,1.07) | 0.90 (0.75,1.09) |
| <60 | 227 | 69 | 1.04 (0.77,1.41) | 1.13 (0.84,1.54) |
| P for trend |  |  | 0.7258 | 0.8796 |
| Per 10-unit decrease |  |  | 1.02 (0.96,1.08) | 1.03 (0.97,1.09) |
| eGFRcys categories | 6230 | 669 |  |  |
| ≥90 | 1664 | 72 | Ref | Ref |
| 60 to <90 | 3253 | 263 | 1.18 (0.90,1.54) | 1.18 (0.90,1.54) |
| <60 | 1313 | 334 | 1.73 (1.30,2.30) | 1.73 (1.30,2.31) |
| P for trend |  |  | <0.0001 | <0.0001 |
| Per 10-unit decrease |  |  | 1.15 (1.10,1.21) | 1.15 (1.10,1.21) |
| eGFRcr-cys categories | 6234 | 670 |  |  |
| ≥90 | 2316 | 107 | Ref | Ref |
| 60 to <90 | 3376 | 389 | 1.20 (0.95,1.51) | 1.22 (0.97,1.54) |
| <60 | 542 | 174 | 1.65 (1.24,2.19) | 1.68 (1.26,2.25) |
| P for trend |  |  | 0.0004 | 0.0003 |
| Per 10-unit decrease |  |  | 1.13 (1.07,1.20) | 1.14 (1.08,1.21) |

Model 1: adjusted for age and sex.

Model 2: adjusted for age, sex, living standard, education level, marital status, residence status, smoking status, drinking status, BMI, hypertension, hyperlipidemia, and history of cardiovascular disease.

| **Supplementary Table 3. The association between different eGFR measures and risk of all-cause mortality among participants with diabetes who excluded those participants with each eGFR<15 and eGFR>120 mL/min/1.73m^2^** | | | | |
| --- | --- | --- | --- | --- |
| eGFR, mL/min/1.73m^2^ | N | Events (%) | Model 1 | Model 2 |
|  |  |  | HR (95% CI) | HR (95% CI) |
| eGFRcr categories | 1346 | 215 |  |  |
| ≥90 | 732 | 63 | Ref | Ref |
| 60 to <90 | 522 | 114 | 1.49 (1.06,2.09) | 1.52 (1.08,2.15) |
| <60 | 92 | 38 | 2.31 (1.48,3.61) | 2.55 (1.62,4.02) |
| P for trend |  |  | 0.0002 | <0.0001 |
| Per 10-unit decrease |  |  | 1.14 (1.05,1.24) | 1.17 (1.07,1.27) |
| eGFRcys categories | 1328 | 213 |  |  |
| ≥90 | 388 | 24 | Ref | Ref |
| 60 to <90 | 630 | 79 | 1.48 (0.93,2.36) | 1.42 (0.88,2.29) |
| <60 | 310 | 110 | 2.72 (1.66,4.43) | 2.90 (1.76,4.78) |
| P for trend |  |  | <0.0001 | <0.0001 |
| Per 10-unit decrease |  |  | 1.70 (1.35,2.15) | 1.32 (1.21,1.43) |
| eGFRcr-cys categories | 1337 | 214 |  |  |
| ≥90 | 493 | 27 | Ref | Ref |
| 60 to <90 | 680 | 119 | 2.03 (1.31,3.14) | 2.01 (1.28,3.14) |
| <60 | 164 | 68 | 3.44 (2.09,5.68) | 3.73 (2.24,6.22) |
| P for trend |  |  | <0.0001 | <0.0001 |
| Per 10-unit decrease |  |  | 1.25 (1.15,1.36) | 1.30 (1.20,1.42) |

Model 1: adjusted for age and sex.

Model 2: adjusted for age, sex, living standard, education level, marital status, residence status, smoking status, drinking status, BMI, hypertension, hyperlipidemia, and history of cardiovascular disease.

| **Supplementary Table 4. The association between eGFRcys or eGFRcr-cys and risk of all-cause mortality among participants without diabetes who had normal kidney function defined by eGFRcr (≥90 mL/min/1.73m^2^)** | | | | |
| --- | --- | --- | --- | --- |
| eGFR, mL/min/1.73m^2^ | N | Events (%) | Model 1 | Model 2 |
|  |  |  | HR (95% CI) | HR (95% CI) |
| eGFRcys categories | 4204 | 262 (6.20) |  |  |
| ≥90 | 1657 | 57 (3.44) | Ref | Ref |
| 60 to <90 | 2272 | 154 (6.78) | 1.28 (0.94,1.74) | 1.29 (0.94,1.77) |
| <60 | 275 | 51 (18.55) | 2.01 (1.34,3.02) | 2.07 (1.37,3.13) |
| P for trend |  |  | 0.0011 | 0.0009 |
| Per 10-unit decrease |  |  | 1.17 (1.08,1.26) | 1.15 (1.06,1.25) |
| eGFRcr-cys categories |  |  |  |  |
| ≥90 | 2456 | 96 (3.91) | Ref | Ref |
| 60 to <90 | 1741 | 164 (9.42) | 1.29 (0.99,1.68) | 1.26 (0.96,1.65) |
| <60 | 7 | 2 (28.57) | NA* | NA* |
| P for trend |  |  | 0.0381 | 0.0681 |
| Per 10-unit decrease |  |  | 1.24 (1.11,1.40) | 1.23 (1.09,1.38) |

Model 1: adjusted for age and sex.

Model 2: adjusted for age, sex, living standard, education level, marital status, residence status, smoking status, drinking status, BMI, hypertension, hyperlipidemia, and history of cardiovascular disease.

*The number of cases were too small for the analysis; NA=not applicable.

| **Supplementary Table 5. The association between eGFRcys or eGFRcr-cys and risk of all-cause mortality among participants with diabetes who had normal kidney function defined by eGFRcr (≥90 mL/min/1.73m^2^)** | | | | |
| --- | --- | --- | --- | --- |
| eGFR, mL/min/1.73m^2^ | N | Events (%) | Model 1 | Model 2 |
|  |  |  | HR (95% CI) | HR (95% CI) |
| eGFRcys categories | 841 | 63 (7.50) |  |  |
| ≥90 | 391 | 15 (3.84) | Ref | Ref |
| 60 to <90 | 396 | 37 (9.34) | 2.16 (1.18,3.97) | 2.36 (1.25,4.47) |
| <60 | 54 | 11 (20.37) | 3.70 (1.59,8.61) | 3.84 (1.58,9.37) |
| P for trend |  |  | 0.0012 | 0.0012 |
| Per 10-unit decrease |  |  | 1.39 (1.21,1.60) | 1.42 (1.23,1.66) |
| eGFRcr-cys categories |  |  |  |  |
| ≥90 | 541 | 24 (4.44) | Ref | Ref |
| 60 to <90 | 298 | 38 (12.75) | 2.37 (1.39,4.04) | 2.42 (1.38,4.22) |
| <60 | 2 | 1 (50.00) | NA* | NA* |
| P for trend |  |  | 0.0006 | 0.0006 |
| Per 10-unit decrease |  |  | 1.59 (1.30,1.94) | 1.64 (1.33,2.01) |

Model 1: adjusted for age and sex.

Model 2: adjusted for age, sex, living standard, education level, marital status, residence status, smoking status, drinking status, BMI, hypertension, hyperlipidemia, and history of cardiovascular disease.

*The number of cases were too small for the analysis; NA=not applicable.

| **Supplementary Table 6. The association between eGFRs and risk of all-cause mortality among individuals without prediabetes** | | | | |
| --- | --- | --- | --- | --- |
| eGFR, mL/min/1.73m^2^ | N | Events (%) | Model 1 | Model 2 |
|  |  |  | HR (95% CI) | HR (95% CI) |
| eGFRcr categories | 3331 | 340 (10.21) |  |  |
| ≥90 | 2071 | 133 (6.42) | Ref | Ref |
| 60 to <90 | 1153 | 173 (15.00) | 0.83 (0.64,1.08) | 0.88 (0.67,1.15) |
| <60 | 107 | 34 (31.78) | 1.17 (0.77,1.80) | 1.31 (0.84,2.04) |
| P for trend |  |  | 0.9198 | 0.6936 |
| Per 10-unit decrease |  |  | 1.00 (0.92,1.09) | 1.03 (0.94,1.12) |
| eGFRcys categories |  |  |  |  |
| ≥90 | 900 | 35 (3.89) | Ref | Ref |
| 60 to <90 | 1711 | 131 (7.66) | 1.19 (0.81,1.73) | 1.18 (0.80,1.73) |
| <60 | 720 | 174 (24.17) | 1.69 (1.13,2.52) | 1.78 (1.18,2.68) |
| P for trend |  |  | 0.0023 | 0.0007 |
| Per 10-unit decrease |  |  | 1.13 (1.06,1.21) | 1.14 (1.06,1.22) |
| eGFRcr-cys categories |  |  |  |  |
| ≥90 | 1264 | 49 (3.88) | Ref | Ref |
| 60 to <90 | 1787 | 211 (11.81) | 1.30 (0.93,1.81) | 1.33 (0.95,1.87) |
| <60 | 280 | 80 (28.57) | 1.50 (0.99,2.28) | 1.62 (1.06,2.49) |
| P for trend |  |  | 0.0622 | 0.0271 |
| Per 10-unit decrease |  |  | 1.12 (1.03,1.20) | 1.14 (1.05,1.23) |

Model 1: adjusted for age and sex.

Model 2: adjusted for age, sex, living standard, education level, marital status, residence status, smoking status, drinking status, BMI, hypertension, hyperlipidemia, and history of cardiovascular disease.

| **Supplementary Table 7. The association between eGFRs and risk of all-cause mortality among individuals with prediabetes** | | | | |
| --- | --- | --- | --- | --- |
| eGFR, mL/min/1.73m^2^ | N | Events (%) | Model 1 | Model 2 |
|  |  |  | HR (95% CI) | HR (95% CI) |
| eGFRcr categories | 3664 | 337 (9.20) |  |  |
| ≥90 | 2133 | 129 (6.05) | Ref | Ref |
| 60 to <90 | 1370 | 169 (12.34) | 0.90 (0.69,1.17) | 0.89 (0.68,1.16) |
| <60 | 161 | 39 (24.22) | 1.03 (0.68,1.54) | 1.10 (0.73,1.66) |
| P for trend |  |  | 0.8384 | 0.9806 |
| Per 10-unit decrease |  |  | 1.06 (0.98,1.14) | 1.06 (0.98,1.15) |
| eGFRcys categories |  |  |  |  |
| ≥90 | 1033 | 41 (3.97) | Ref | Ref |
| 60 to <90 | 1876 | 132 (7.04) | 1.11 (0.78,1.60) | 1.10 (0.76,1.58) |
| <60 | 755 | 164 (21.72) | 1.73 (1.17,2.57) | 1.66 (1.11,2.48) |
| P for trend |  |  | 0.0009 | 0.0024 |
| Per 10-unit decrease |  |  | 1.15 (1.08,1.23) | 1.14 (1.07,1.22) |
| eGFRcr-cys categories |  |  |  |  |
| ≥90 | 1365 | 61 (4.47) | Ref | Ref |
| 60 to <90 | 1945 | 178 (9.15) | 1.05 (0.77,1.43) | 0.99 (0.72,1.37) |
| <60 | 354 | 98 (29.08) | 1.63 (1.10,2.40) | 1.54 (1.04,2.28) |
| P for trend |  |  | 0.0075 | 0.0159 |
| Per 10-unit decrease |  |  | 1.14 (1.06,1.23) | 1.13 (1.05,1.22) |

Model 1: adjusted for age and sex.

Model 2: adjusted for age, sex, living standard, education level, marital status, residence status, smoking status, drinking status, BMI, hypertension, hyperlipidemia, and history of cardiovascular disease.

| **Supplementary Table 8. IDI, NRI, and Changes in C index for composite all-cause death outcome for all three eGFR measures** | | | |
| --- | --- | --- | --- |
| Comparator | Addition | Diabetes (95%CI) | Non-diabetes (95%CI) |
| IDI |  | | |
| Model 2 | + eGFRcr | 0.7% (0.2%,2.1%) | 0.1% (0.05%,0.3%) |
| Model 2 | + eGFRcys | 3.6% (1.6%,7.0%) | 0.9% (0.6%,1.4%) |
| Model 2 | +eGFRcr-cys | 2.8% (1.4%,5.6%) | 0.7% (0.5%,1.1%) |
| NRI |  | | |
| Model 2 | + eGFRcr | 7.6% (-5.1%,14.9%) | -0.9% (-0.8%,5.4%) |
| Model 2 | + eGFRcys | 20.1% (10.6%,27.9%) | 12.1% (7.2%,15.8%) |
| Model 2 | +eGFRcr-cys | 19.2% (7.4%,25.3%) | 8.2% (3.2%,14.0%) |
| Changes in C index |  | | |
| Model 2 | + eGFRcr | 0.0107 (-0.0028,0.0218) | 0.0002 (-0.0005,0.0010) |
| Model 2 | + eGFRcys | 0.0340 (0.0029,0.0628) | 0.0037 (-0.0003,0.0088) |
| Model 2 | +eGFRcr-cys | 0.0306 (0.0023,0.0559) | 0.0028 (0.0001,0.0065) |

eGFRcr-cys, estimated glomerular filtration rate based on serum creatinine and serum cystatin C; eGFRcr, estimated glomerular filtration rate based on serum creatinine; eGFRcys, estimated glomerular filtration rate based on serum cystatin C; IDI, Integrated discrimination


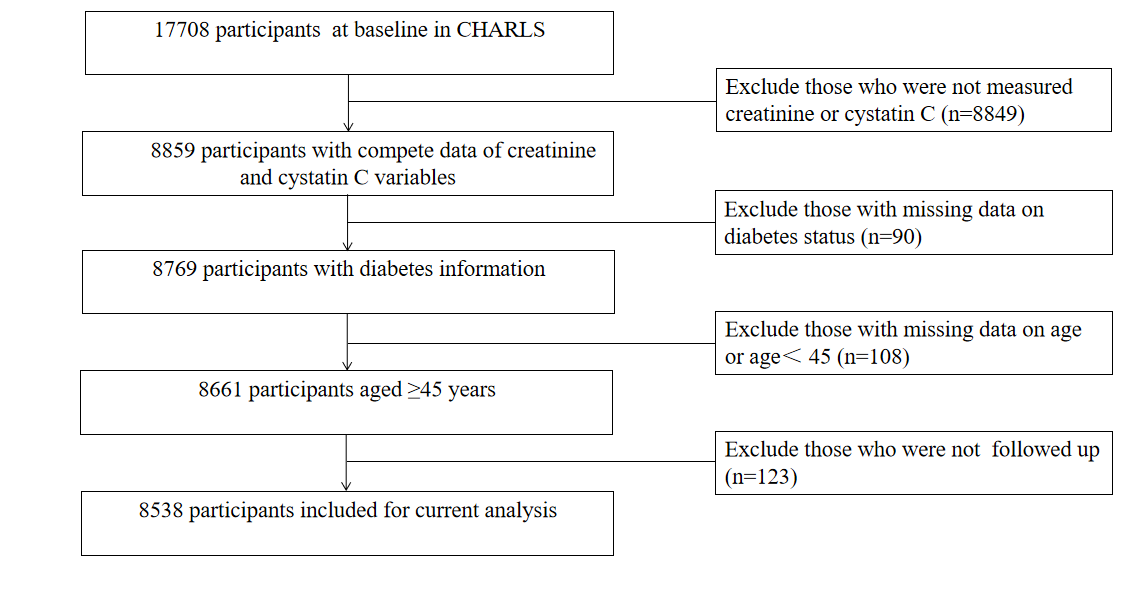


**Supplementary Figure 1. The flow chart of participant selection.**
